# The association between smoking and smokeless tobacco use with dental caries among Pakistani patients

**DOI:** 10.1186/s12903-024-04508-y

**Published:** 2024-06-24

**Authors:** Muhammad Azad Khan, Tippanart Vichayanrat, Yaowaluk Ngoenwiwatkul

**Affiliations:** 1 Master of Science in Dentistry Program, Faculty of Dentistry, Mahidol University, 6 Yothi Street, Rajthevee, Bangkok, 10400 Thailand; 2 Community Dentistry Department, Sandeman Provincial Civil Hospital, Anscomb Road, Quetta, 87300 Pakistan; 3 Department of Community Dentistry, Faculty of Dentistry, Mahidol University, 6 Yothi Street, Rajthevee, Bangkok, 10400 Thailand

**Keywords:** Tobacco, Dental caries, Adult, Pakistan, Oral health

## Abstract

**Background:**

Tobacco use is widely recognised as a significant risk factor for oral cancer and periodontal diseases. However, the relationship between various types of tobacco use and dental caries has been inconsistent. This study aimed to determine the association between smoking and smokeless tobacco and dental caries among patients in a tertiary care hospital in Quetta, Pakistan.

**Methods:**

This cross-sectional study was conducted from November 2020 to March 2021 among patients in a tertiary care hospital in Quetta, Pakistan. Oral examinations and interviews were performed according to the WHO Oral Health Survey basic methods (5th edition) to determine dental caries status, tobacco use, and oral health behaviours. The odds ratio and binary logistic regression were analysed to assess the association between the types (smoking tobacco, smokeless tobacco, and use of both types), duration, and frequency of tobacco use and high caries level (DMFT ≥ 5).

**Results:**

Four hundred participants aged 18–65 were included, and 67.8% were tobacco users. Use of both smoking and smokeless tobacco (aOR = 14.7, 95%CI = 1.87–115.96, *p* = 0.011), smokeless tobacco only (aOR = 5.90, 95%CI = 1.89–18.37, *p* = 0.002), and smoking only (aOR = 2.18, 95%CI = 1.23–3.88, *p* = 0.008) were associated with significantly increased risk of high caries. Using tobacco for longer periods and more frequently daily also significantly increases the risk of high caries.

**Conclusion:**

Smoking and smokeless tobacco are significantly associated with high dental caries after adjusting for other factors. Due to the high severity of dental caries and the high prevalence of tobacco use among Pakistani patients in this study, it is highly recommended to strengthen policies aimed at reducing tobacco usage, including smokeless forms.

**Supplementary Information:**

The online version contains supplementary material available at 10.1186/s12903-024-04508-y.

## Background

Tobacco remains a significant public health concern, with more than 8 million deaths attributed to its use annually, with low-to-middle-income countries experiencing the highest burden [1]. Various tobacco types are responsible for many non-communicable diseases, including cardiovascular diseases, respiratory diseases, and cancers in multiple organs [2]. The economic cost of smoking is reported to be up to 5.7% of global health expenditure, of which 40% is attributed to developing countries [3]. In Pakistan, tobacco consumption results in substantial economic burdens on healthcare, with the combined costs of three major smoking-related diseases (cancer, cardiovascular disease, and respiratory disease) totalling PKR 437.76 billion, representing 71% of the overall economy and health costs associated with tobacco [4]. The age-standardised prevalence of tobacco use in Pakistan was 13.4%, and males were 7 times more likely to use tobacco than females [5]. The Global Adult Tobacco Survey found that 40.3% of current tobacco users use smokeless tobacco, with naswar being the most commonly used smokeless tobacco product at 5.1% in Pakistan [6].

Tobacco use is widely recognised as a significant risk factor for oral cancer, periodontal diseases, peri-implantitis, precancerous lesions, caries, alveolar osteitis and halitosis [7–9]. The form in which tobacco is consumed influences the oral cavity varies. Smoking tobacco creates combustion products that enter through the oral cavity, while smokeless tobacco is generally consumed orally and often remains in contact with the oral tissues for a prolonged period. There are many forms of smokeless tobacco, such as chewing tobacco, snuff, snus, and dissolvable tobacco products, with some forms used primarily in South Asia and the Pacific, such as betel quid (generally consists of betel leaf, areca nut, and slaked lime, and often added with tobacco). In Pakistan, smokeless tobacco use is common, including the use of *naswar* (moist tobacco dip containing slaked lime, ash, cotton or sesame oil, menthol, flavoring and coloring agents, and water), *gutka* (crushed betel nut, tobacco, and sweet or savoury flavourings), and *khaini* (betal quid with power tobacco, slaked lime, areca nut, catechu, tombol leaf, and powdered khat) [10]. Most smokeless tobacco use involves placing the product between the gum and the cheek or lip, sucking, and chewing for 15 to 30 min. For smoking tobacco, Bidi (also known as Beedi) is a hand-rolled cigarette filled with tobacco flake and commonly wrapped in a tendu leaf tied with a string or adhesive at one end.

The relationship between caries and different forms of tobacco use has been subject to debate and varying conclusions [11, 12]. A recent study in India found that tobacco chewers had a higher incidence of dental caries compared to tobacco smokers, and those using gutka had higher DMFT scores compared to other forms of tobacco use [12]. Nonetheless, a previous study indicated that the risk of dental caries increased with frequency and duration of smoking but no significant association in DMFT among tobacco chewers with frequency and duration [11]. A systematic review and meta-analysis indicated a significant relationship between smoking and increased dental caries risk; however, the study did not include exposure to smokeless tobacco products [13]. Since epidemiological studies on caries and tobacco in Pakistan have been scarce, and the association between dental caries and various types of tobacco is still controversial, it is pertinent to explore the prevalence of tobacco use and its relation to dental caries in this area. Therefore, this study aimed to determine the prevalence of tobacco use, including smoking and smokeless tobacco, and explore the relationship between various tobacco use and dental caries among patients at a tertiary care hospital in Quetta, Pakistan.

## Materials and methods

### Study design and participants

This cross-sectional study was conducted from November 2020 to March 2021 at Sandeman Provincial Civil Hospital, Quetta, Pakistan. Quetta is the provincial capital and largest city in Balochistan, which is the largest province in Pakistan. The province is largely underdeveloped and has the highest poverty rate, infant and maternal mortality rate, and lowest literacy rate in Pakistan [14]. The inclusion criteria comprised patients aged 18–65 who lived in Balochistan province, visited the Dental Department, and were willing to participate. The exclusion criteria were those with a mental illness, who were physically dependent, who had any systemic disease, or who were foreigners from another country or province.

## Sampling and data collection

The participants were recruited as convenience samples. Patients who visited the Sandeman Provincial Civil Hospital at the Dental Department were invited to participate in the study if they met the inclusion criteria. After the medical history was taken and the researcher explained the objectives of the study, the patients with a mental illness, were physically dependent, had any systemic disease, or were from other provinces were excluded. The informed consents were obtained before the interview and oral examination. The oral examination and interview were performed according to the WHO Oral Health Basic Method (5th edition) and modified WHO Oral Health questionnaire for adults [15].

### Questionnaire development

The questionnaire in this study was modified from WHO Oral Health Questionnaire for Adults [15] to assess the types, frequency, and years of tobacco use. Since the original questionnaire asked only one question about the frequency of tobacco use, the modified questionnaire included more details of tobacco use as follows. The types of tobacco use were added to defined as smoking tobacco (cigarette, sheesha, bidi), and smokeless tobacco (naswar and gutka). The frequency of tobacco and the period of tobacco use were also asked. In addition, the participants were asked about knowledge of the harmful effects of tobacco use, tooth cleaning, and sweet consumption behaviours according to the original WHO Oral Health Questionnaire for Adults (Appendix 1).

The modified questionnaire was tested for content validity by two dental public health specialists until all the questions were relevant to the research objectives. Then, the questionnaire was pilot-tested in samples and revised to ensure the understanding and meaning in the local language before data collection. After the revision, the question about the frequency of tobacco use was changed to make it easier to understand by asking participants to indicate how often they use tobacco (occasional, many times a week, or every day). If they use tobacco every day, then the follow-up question on how many times a day was asked. The final questionnaire validity was more than 0.8 for the content validity index, and each item was relevant or highly relevant to the measured domain [16].

### Oral examination

For oral examination validity and reliability, a dentist was trained for a two-day workshop on oral health survey according to the WHO oral health basic method [15] by the Bureau of Oral Health, Ministry of Public Health, Bangkok, Thailand. The trained dentist performed oral examinations after participants provided consent. Participants were seated on a dental chair and examined using a mouth mirror and WHO probe. The decayed, missing, and filled teeth (DMFT) index was assessed according to the 2013 WHO Oral Health Assessment Form for Adults [15] (Appendix 2). After the oral examination, the dentist conducted the interview questionnaire, and another trained dentist helped fill out the questionnaire form.

### Variable measures

Since only one person has no dental caries (DMFT = 0), the outcome variables were categorised as high (DMFT ≥ 5) and low (DMFT < 5) levels of caries. Tobacco use was measured for type, frequency, and duration. Types of tobacco use were categorised into no tobacco use, smoking, smokeless, and both types of tobacco. The smoking tobacco included cigarettes, bidi, and sheesha. The smokeless tobacco included naswar and gutka. The frequency was categorised as less than 5 times daily and ≥ 5 times daily. The duration of tobacco use was categorised as ≤ 10 years and more than 10 years.

The sociodemographic variables were gender, age, education, income, and living location. Age was collected in years, then divided into 3 groups: young (18–34 years), middle-aged (35–50 years), and pre-ageing (51–65 years). Education was categorised as no education, primary school (completed 6 years), secondary school (completed 10 years), and high school (completed 12 years) or above. Location was defined as living in Quetta City or outside Quetta City. Income was categorised as low (< $1,000), middle ($1,000–10,000), and high (> $10,000)*($1* = *154PKR)*. The behavioural variables were tooth cleaning and sweet consumption. Tooth cleaning frequency was categorised as never, sometimes, or once daily. Sweet consumption (i.e., biscuits, cakes, cream cakes, sweets, candy) was categorised into low frequency (never/once a week) and high frequency (several times a week, every day, several times a day). In addition, the participants were asked whether they knew about the harmful effects of tobacco use (yes or no) (Appendix 1).

### Data analysis

The data were analysed using SPSS version 24.0 for descriptive and inferential statistical analysis. For bivariate analysis, the socio-demographic variables (age, gender, education, income and location), knowledge, behavioural factors, and tobacco use associated with high caries (DMFT ≥ 5) were analysed using crude odds ratio. For the multivariate analysis, binary logistic regression was conducted to explain the relationships between types of tobacco use (no tobacco use, smoking tobacco, smokeless tobacco, or both types of tobacco) and the high caries level (DMFT ≥ 5), controlling for gender, age, education, knowledge, tooth cleaning frequency, and sweet consumption. For the crude and adjusted odds ratios, age was divided into ≤ 50 years old and > 50 years old, income was divided into < 10,000 USD and ≥ 10,000 USD, and education was divided into high school or above and lower than high school.

### Ethical considerations

This research followed the Declaration of Helsinki. The Ethical Review Board of Bolan Medical College approved the study protocol for use at Sandeman Provincial Civil Hospital, Quetta, Pakistan. Written informed consent was obtained from all participants. The research proposal was reviewed and approved by the ethical committee of the Research Centre, Bolan University of Medical and Health Sciences, Quetta, Pakistan (No. BUHMS/Reg/2020/4241).

## Results

A total of 400 participants aged 18–65 (mean age = 42.2 ± 11.4 years) were included; 210 (52.5%) were males, 116 (29%) completed high school or further education, and 167 (42%) were unemployed. Additionally, 271 (67.8%) were tobacco users, 399 (99.8%) persons had dental caries experience (99.8%), and 308 (77%) of the participants had DMFT ≥ 5.

Table 1 describes the prevalence of smoking and smokeless tobacco use. Among 271 tobacco users, 32 (11.8%) were dual users, 77 (28.4%) used smokeless tobacco only, and 162 (59.8%) used smoking tobacco only. In addition, 75% of males and 59% of females used tobacco products. Patients outside Quetta City used more tobacco than those who lived inside the city. Tobacco use increased with age, with 84% of the pre-ageing group and 62% of the younger group being tobacco users. More participants with no education were tobacco users (89%) than those who completed primary (78%), secondary (56%), or higher education (51%). All low-income patients used tobacco, while 64% and 86% of middle-and high-income patients were tobacco users, respectively. The cigarette was the most popular type of tobacco used; however, naswar use was more common among low-income and low-education participants.

**Table 1:** Prevalence of smoking and smokeless tobacco use (can choose more than 1 type of tobacco product) (*N* = 400)

Characteristics	N	Tobacco Use (%)	Smoking Tobacco			Smokeless tobacco	
			Cigarette	Bidi	Sheesha	Naswar	Gutka
Gender							
Male	210	158 (75%)	101 (48%)	20 (10%)	3 (1%)	54 (26%)	11 (5%)
Female	190	113 (59%)	46 (24%)	30 (16%)	9 (5%)	38 (20%)	14 (7%)
Location							
Quetta City	291	172 (59%)	100 (34%)	24 (8%)	7 (2%)	52 (18%)	13 (4%)
Out of Quetta City	109	99 (91%)	47 (43%)	26 (24%)	5 (5%)	40 (37%)	12 (11%)
Age Group							
Young	103	64 (62%)	34 (33%)	10 (10%)	6 (6%)	19 (18%)	10 (10%)
Middle Aged	196	122 (62%)	72 (37%)	19 (10%)	5 (3%)	39 (20%)	6 (3%)
Pre-Aging	101	85 (84%)	41 (41%)	21 (21%)	1 (1%)	34 (34%)	9 (9%)
Education							
No Education	84	75 (89%)	24 (29%)	23 (27%)	1 (1%)	43 (51%)	14 (17%)
Primary School	114	89 (78%)	49 (43%)	11 (10%)	3 (3%)	33 (29%)	4 (4%)
Secondary School	86	48 (56%)	32 (37%)	10 (12%)	5 (6%)	4 (5%)	3 (3%)
High School or Above	116	59 (51%)	42 (36%)	6 (5%)	3 (3%)	12 (10%)	4 (3%)
Income							
Low	24	24 (100%)	8 (33%)	6 (25%)	0 (0%)	13 (54%)	4 (17%)
Middle	348	223 (64%)	119 (34%)	41 (12%)	12 (3%)	73 (21%)	21 (6%)
High	28	24 (86%)	20 (71%)	3 (11%)	0 (0%)	6 (21%)	0 (0%)
Total	400	271 (67.8%)	147 (37%)	50 (13%)	12 (3%)	92 (23%)	25 (6%)

Table 2 demonstrates the crude odds ratio of high caries level (DMFT ≥ 5) related to sociodemographic variables, knowledge, oral health behaviours, and tobacco use. Age, education, location, knowledge, teeth-cleaning frequency, sweet consumption, types, duration, and frequency of tobacco use were significantly associated with dental caries status. Middle-aged (odds ratio = 2.28, *p* = 0.002) and pre-ageing groups (odds ratio = 8.80, *p* < 0.001) were more likely to have high dental caries than younger participants, respectively. Participants with lower education levels had higher levels of caries than those with higher education levels (odds ratio = 6.29, *p* < 0.001). Participants who lived outside Quetta City had higher dental caries levels than those who lived in Quetta City (odds ratio = 6.91, *p* = 0.008). Participants who were unaware of the harmful effects of tobacco had higher levels of caries than those who were aware (odds ratio = 6.91, *p* = 0.008). Participants who cleaned their teeth less than once a day had higher caries than those who cleaned their teeth at least once daily (odds ratio = 3.94, *p* < 0.001). Participants who consumed sweets at high frequencies were more likely to have high dental caries (odds ratio = 1.87, *p* = 0.013). For the types of tobacco use, using both types of tobacco (odds ratio = 24.54, *p* = 0.002), using smokeless tobacco only (odds ratio = 14.45, *p* < 0.001), and smoking tobacco only (odds ratio = 3.48, *p* < 0.001), increased the high caries levels than non-tobacco users, respectively. Participants who used tobacco for less than 10 years (odds ratio = 2.34, *p* = 0.004) and more than 10 years (odds ratio = 11.88, *p* < 0.001) had increased higher caries levels than non-tobacco users. Participants who used tobacco less than 5 times daily (odds ratio = 3.17, *p* = 0.045) and more than 5 times daily (odds ratio = 5.62, *p* < 0.001) had increased higher caries levels than non-tobacco users.

**Table 2:** Bivariate analysis of the association between sociodemographic variables, knowledge, oral health behaviours, tobacco use and high caries level (DMFT ≥ 5)

		High caries (DMFT ≥ 5)		*p*-value
		Crude OR	95% CI	
Age	Young	Reference		
	Middle-aged	2.28	1.36—3.83	0.002
	Pre-ageing	8.80	3.74—21.05	< 0.001
Gender	Female	Reference		
	Male	1.02	0.64 -1.62	0.943
Education	High school or above	Reference		
	Lower than high school	6.29	3.54 -11.16	< 0.001
Income	≥ 10,000 USD	Reference		
	< 10,000 USD	1.37	0.58—3.23	0.469
Location	Quetta city	Reference		
	Outside Quetta city	2.49	1.34—4.61	0.004
Know harmful effects of tobacco	Yes	Reference		
	No	6.91	1.64—29.14	0.008
Tooth cleaning frequency	At least once a day	Reference		
	Less than once a day	3.94	2.10 -7.40	< 0.001
Sweet consumption	Low frequency	Reference		
	High frequency	1.87	1.14—3.07	0.013
Type of tobacco	None	Reference		
	Both types	24.54	3.25 -185.26	0.002
	Smokeless tobacco only	14.45	4.98—41.90	< 0.001
	Smoking tobacco only	3.48	2.06—5.90	< 0.001
Duration of tobacco use	None	Reference		
	≤ 10 years	2.34	1.31—4.18	0.004
	> 10 years	11.88	5.88—23.97	< 0.001
Frequency of tobacco use	None	Reference		
	< 5 times daily	3.17	1.00—9.99	0.045
	≥ 5 times daily	5.62	3.37—9.38	< 0.001

Table 3 presents the multivariate analysis to determine the factors associated with having high caries (DMFT >  = 5). Binary logistic regression was performed to ascertain the association of sociodemographic variables, behaviour, knowledge, and tobacco types on the likelihood of high dental caries level (DMFT ≥ 5) versus low dental caries level (DMFT < 5). The logistic regression model was statistically significant, chi-square (df = 10, N = 400) = 98.34, *p* < 0.001. The model explained 33.0% (Nagelkerke R^2^) of the variance and correctly classified 79.5% of cases. After controlling for sociodemographic variables, behaviour, and knowledge, the use of both smoking and smokeless tobacco (aOR = 19.12, 95%CI = 2.33–156.97), smokeless tobacco only (aOR = 7.40, 95%CI = 2.25–24.32), and smoking tobacco only (aOR = 2.97, 95%CI = 1.50–5.20) was associated with high caries level. Age (aOR = 3.33, 95%CI = 1.40–7.90), education (aOR = 3.21, 95%CI = 1.64–6.24), and sweet consumption (aOR = 2.13, 95%CI = 1.17–3.86) were also significantly associated with high dental caries.

**Table 3:** Multivariate logistic regression analysis to determine the association of tobacco use and high caries level (DMFT > 5)

Variables	aOR	95% CI
Sociodemographic		
Age (≥ 50 years old)	3.33**	1.40—7.90
Education (lower than high school)	3.21**	1.64—6.24
Gender (Male)	1.25	0.71—2.19
Location (outside Quetta city)	1.03	0.21—5.18
Behaviour		
Tooth cleaning (< once a day)	1.03	0.49—2.16
Sweet consumption (high frequency)	2.13*	1.17—3.86
Knowledge (do not know harmful effect of tobacco)	1.04	0.21–5.18
Tobacco types^a^		
Use both types	19.12**	2.33—156.97
Smokeless tobacco only	7.40**	2.25—24.32
Smoking tobacco only	2.79**	1.50—5.20

Reference group (age = less than 50 years old, education = high school or above, gender = female, tooth cleaning = at least once a day, Location = Quetta City, Sweet consumption = low frequency, Knowledge = yes)

*aOR* adjusted odds ratio, *CI* Confidence interval

Dependent variable: DMFT level (1 = high caries DMFT ≥ 5; 0 = low dental caries (DMFT < 5)

^*^p < 0.05, **p < 0.01, ***p < 0.001

^a^No tobacco use as a reference

## Discussion

This study aimed to investigate the relationships between different forms of tobacco consumption and the dental caries experience. The findings added to the evidence that tobacco use, regardless of how it is used, is associated with severe caries status. The dose–response relationship of tobacco and dental caries is also significant in terms of frequency of use and years of using tobacco. Our findings revealed that dental caries escalated progressively from individuals who did not use tobacco to those who exclusively smoked tobacco, then to those who exclusively used smokeless tobacco, reaching the highest among those who used both smoking and smokeless tobacco. This gradient proved significant under multivariate analysis since the odds of having high dental caries increased from 2.79 in the smoking group to 7.40 in the smokeless group and 19.12 in dual users after adjusting for age, education, and sweet consumption.

The relationship between dental caries and smoking tobacco in this study was similar to previous studies that showed the caries development in smokers was significantly higher than that of non-smokers [17, 18]. Smoking tobacco may contribute to dental caries by promoting cariogenic activity among oral microorganisms, such as enhancing the growth of *Streptococcus sanguinis* and increasing the adhesion of *Streptococcus mutans* and *Candida albicans* to the acquired pellicle [19]. Furthermore, smoking reduces the buffer capability of saliva and alters its chemical components, promoting the formation of a caries-susceptible environment. A laboratory experiment demonstrated that nicotine stimulates the biofilm formation and metabolic activity of oral pathogens, such as *Streptococcus mutans*, thereby contributing to dental caries [20].

The results of the present study supported that smokeless tobacco users were more likely to have higher caries experience than smoking tobacco users. Our study corresponds with the study by Doddawad et al., which indicated a higher presence of caries among tobacco chewers than among smokers [12]. This finding may be due to the characteristics of the smokeless tobacco, which contains act as cariogenic agents, and the methods of using smokeless tobacco by chewing or sucking in the oral cavity for a period of time. In addition, the smokeless tobacco users in the present study consumed tobacco daily with high frequency, which explained the high odds ratio observed that differed from the results of a previous study, where no association between smokeless tobacco and dental caries was found [11]. The term ‘smokeless tobacco’ can refer to various smokeless forms. In this study, the smokeless forms included chewing and dipping tobacco such as naswar and gutka. This type of tobacco is typically placed on the mandibular sulcus and sucked slowly and often contains flavours and sweetening agents. Other studies involving smokeless tobacco may refer to snuff, a powder form of loose tobacco that a person sniffs or inhales through the nostrils, or snus or Swedish snus, which claim to contain neglectable amounts of fermentable carbohydrates and have a high pH value and low level of tobacco-specific nitrosamines [21]. Therefore, the ingredients of smokeless tobacco are processed and used in very different ways, and there may be different levels of health risks. Future studies should be conducted to validate the impact of different types of smokeless tobacco on dental caries across diverse populations, taking into account and controlling for potential influencing factors.

Although decreased smoking tobacco use was reported following the implementation of the Framework Convention on Tobacco Control (FCTC) policies between 2008 and 2016, smokeless tobacco consumption began to increase due to the increasing number of low- and middle-income households [22]. Our findings support this pattern, as smokeless tobacco, such as naswar, was more commonly used among low-income participants. A high proportion of naswar users were low-income participants (54%), as naswar is less expensive than other tobacco products. Additionally, smokeless tobacco such as naswar is not regulated in Pakistan and is freely available compared to cigarettes, which are more controlled [23]. In contrast, high-income participants used cigarettes more than other tobacco products.

Findings from the Global Adult Tobacco Survey 2014 indicated that 19.1% of adults were currently using tobacco products in Pakistan [24]. In contrast, a study among women in Karachi found that 53.8% had at least one person in their home who used tobacco, while 42.3% of them used smokeless tobacco, and 18% used smoked tobacco [25]. However, in our study, 67.8% of adult patients were tobacco users. With the lack of the current situation of tobacco use in Pakistan, especially in Quetta city, which is generally underdeveloped, it was difficult to compare our findings to the general Pakistani population. Nonetheless, the present data indicated that patients who visited dental healthcare services might be associated with a higher rate of tobacco consumption. A study in Karachi discovered that 49.8% of young adult patients (aged 15–30) and 52.4% of adult patients visiting family medicine clinics used some form of smokeless tobacco [26]. One explanation is that people visiting healthcare facilities might have more health problems than the general population, which may be related to high tobacco use behaviours. Therefore, the prevalence of tobacco consumption must be interpreted with caution when comparing study settings and the representativeness of different population groups.

Tobacco consumption in Pakistan is a significant public health concern that requires effective interventions at the population and policy level to solve. Rising cigarette prices in 2019 prompted 29% of smokers to switch to smokeless, with 13% seriously considering quitting, demonstrating the potential of taxation to discourage smoking in Pakistan [27]. However, in contrast to smoking, measures aimed at regulating smokeless tobacco substantially fall behind [28]. While dental professionals should incorporate anti-smoking activities into their caries prevention strategies, clinic-level smoking cessation programmes are hard to implement if national policies and campaigns are not reinforced. Future studies on effective policy and interventions are urgent needed to reduce the tobacco consumption especially smokeless tobacco in Pakistan.

This study has several limitations. This research was conducted during the global COVID-19 pandemic, which interrupted the data collection phase following the closure of the hospital. This may have increased the chance that patients at a high risk of developing caries were included in the study. Results from hospital-based surveys, particularly those conducted during COVID-19, should be interpreted with caution. Furthermore, the tobacco use and caries status among the participants from Balochistan included in this study were high severity and unique due to resource scarcity in the province. Therefore, the sample used in this study may not be comparable to populations in other areas of Pakistan.

## Conclusion

Use of both smoking and smokeless tobacco, smokeless tobacco only and smoking only, were associated with a significantly increased risk of high caries level. Long-term use of tobacco and frequent daily use were notably associated with a higher risk of dental caries. Due to the severity of dental caries and the high prevalence of tobacco use among Pakistani patients in this study, implementing policies to reduce tobacco consumption, including smokeless forms, is strongly recommended.

### Supplementary Information

Supplementary Material 1.

Supplementary Material 2.

## Data Availability

The datasets used and analysed during the current study are available from the corresponding author on reasonable request.
