# Supplementary material for: The association between smoking and smokeless tobacco use with dental caries among Pakistani patients
Source: BMC Oral Health. 2024 Jun 24;24:723. doi: 10.1186/s12903-024-04508-y (PMC11197218; doi:10.1186/s12903-024-04508-y)
Supplement: Supplementary file 1 — Supplementary Material 1. [file 12903_2024_4508_MOESM1_ESM.pdf]

Appendix A
Questionnaire for assessing oral health behaviors and use of different tobacco products for Adults

Patient ID: ……………….. **Date: …..……………**

	**Name**	**Sex**	**Age (in years)**
**1.**	--------------------------	☐ 1 ☐ 2	--------------------------

---

2. Location

Quetta 1 ☐ Other 2 ☐

---

3. What is your annual income? *($1 = 154PKR)*

Up to $1,000………………………………………… ☐ 1

Between $1,000 to $5,000…………………………….. ☐ 2

Between $6,000 to $10,000………………………………. ☐ 3

Above $10,000…………………………………………… ☐ 4

---

4. What level of education have you completed?

No Education .... ☐ 0

Less than primary school .... ☐ 1

Primary school completed .... ☐ 2

Secondary school completed .... ☐ 3

High school completed .... ☐ 4

College/university completed .... ☐ 5

Postgraduate degree .... ☐ 6

---

5. What is your occupation?

Unemployed .... ☐ 0

Labor/ Daily wager .... ☐ 1

Self Employed .... ☐ 2

Government Employee .... ☐ 3

Private sector Employee .... ☐ 4

---

6. How often do you clean your teeth?

Never .... ☐ 0

Once a month .... ☐ 1

2 -3 times a month .... ☐ 2

Once a week .... ☐ 3

2–6 times a week .... ☐ 4

Once a day .... ☐ 5

Twice or more a day .... ☐ 6

---

7. Do you use any of the following to clean your teeth?

(Read each item)	Yes<br>1	No<br>2
Toothbrush	☐	☐
Wooden toothpicks	☐	☐
Plastic toothpicks?	☐	☐
Thread (dental floss)	☐	☐
Charcoal	☐	☐
Chewstick/miswak	☐	☐

Other .... ☐ ☐

Please specify .... ☐ ☐

7.

	Yes	No
**a)** Do you use toothpaste to clean your teeth	1☐	2☐
**b)** Do you use a toothpaste that contains fluoride?	1 ☐	2 ☐
Don't know		☐ 9

---

8. How long is it since you last saw a dentist?

Less than 6 months .... ☐ 5

6–12 months .... ☐ 4

More than 1 year but less than 2 years .... ☐ 3

2 years or more but less than 5 years .... ☐ 2

5 years or more .... ☐ 1

Never received dental care .... ☐ 0

---

9. Have you ever felt any discomfort or pain in your mouth/ teeth, who do you prefer to seek help from?

A dental care hospital .... ☐ 1

A dental clinic with a specialized doctor .... ☐ 2

A dental technician .... ☐ 3

A quack .... ☐ 4

---

10. How often do you eat or drink any of the following foods, even in small quantities?

(Read each item)	Several times a day 5	Every day 4	Several times a week 3	Once a week 2	Several times a month 1	Seldom / never 0
Fresh fruit / Fruit Juice	☐	☐	☐	☐	☐	☐
Biscuits, cakes, cream cakes	☐	☐	☐	☐	☐	☐

Jam or honey	☐	☐	☐	☐	☐	☐
Chewing gum containing sugar	☐	☐	☐	☐	☐	☐
Sweets/candy	☐	☐	☐	☐	☐	☐
Lemonade, Coca Cola or other soft drinks	☐	☐	☐	☐	☐	☐
Tea/ Coffee with sugar	☐	☐	☐	☐	☐	☐

---

11. Do you use any form of tobacco? If yes, refer to question # 12. If No, refer to question # 17.

	Yes	No
	1 ☐	2 ☐

12. Which form of the following tobacco do you use?

Smoking tobacco

Cigarette ……………………………………………..	1 ☐	2 ☐
Bidi…………………………………………………..	1 ☐	2 ☐
Sheesha……………………………………………….	1 ☐	2 ☐

Smokeless tobacco

Naswar ……………………………………………….	1 ☐	2 ☐
Gutka …………………………………………………	1 ☐	2 ☐
Others (please specify) ……………………………….	1 ☐	2 ☐

---

13. How often do you use any of the above forms of tobacco?

	Every day	Several times a week	Occasional
	3	2	1
Cigarette………………………………	☐	☐	☐
Bidi……………………………………	☐	☐	☐
Sheesha ………………………………..	☐	☐	☐
Naswar…………………………………	☐	☐	☐
Gutka…………………………………..	☐	☐	☐
Others (please specify)………………...	☐	☐	☐

---

14. If you are a daily tobacco user, how many times a day do you use?

	1 to 4 times	5 to 10 times	More than 10 times
	3	2	1
Cigarette……………………………….	☐	☐	☐
Bidi…………………………………….	☐	☐	☐
Sheesha ………………………………..	☐	☐	☐
Naswar…………………………………	☐	☐	☐
Gutka…………………………………..	☐	☐	☐
Others (please specify)………………...	☐	☐	☐

---

15. Since how long have you been using tobacco?

(Please specify in years) …………………………

---

16. What were the factors that influenced you to start using tobacco?

Peer pressure ……………………………… ☐ 1

Occupational stress …………………………... ☐ 2

Domestic stress ………………………………. ☐ 3

For Enjoyment ……………………………….. ☐ 4

☐

Others (Please specify) ……………………..... 5

---

17. Do you have any knowledge of harmful effects of tobacco use on oral health?

Yes 1☐ No ☐

18. How much do you believe in the warning labels on cigarette or snuff packaging?

Do not believe ……………………………………………… ☐ 1

Believe to some extent …………………………………... ☐ 2

Strongly believe ……………………………………………. ☐ 3

---

17. Do you feel any discomfort while using any form of tobacco?

Embarrassment in public ……………………………………. ☐ 1

Family pressure ……………………………………………….. ☐ 2

Non-smoking place/ workplace……………………………... ☐ 3

Any other (please specify) …………………………………….. ☐ 4

---

18. Do you intend or have you ever intended quit tobacco using?

Yes 1☐ No 2☐

Year	Month	Day	Interviewer	District	Country
☐☐	☐☐	☐☐	------------------	----------------	------------------

---
