# Supplementary material for: The association between smoking and smokeless tobacco use with dental caries among Pakistani patients
Source: BMC Oral Health. 2024 Jun 24;24:723. doi: 10.1186/s12903-024-04508-y (PMC11197218; doi:10.1186/s12903-024-04508-y)
Supplement: Supplementary file 2 — Supplementary Material 2. [file 12903_2024_4508_MOESM2_ESM.pdf]

# Appendix B

## World Health Organization Oral Health Assessment Form for Adults, 2013

Crown 45) 60)

| 18 | 17 | 16 | 15 | 14 | 13 | 12 | 11 | 21 | 22 | 23 | 24 | 25 | 26 | 27 | 28 |
|---|---|---|---|---|---|---|---|---|---|---|---|---|---|---|---|
| | | | | | | | | | | | | | | | |
| | | | | | | | | | | | | | | | |

Crown 77) (92)

| 48 | 47 | 46 | 45 | 44 | 43 | 42 | 41 | 31 | 32 | 33 | 34 | 35 | 36 | 37 | 38 |
|---|---|---|---|---|---|---|---|---|---|---|---|---|---|---|---|
| | | | | | | | | | | | | | | | |
| | | | | | | | | | | | | | | | |

**Permanent teeth**

**Status**

0 = Sound
1 = Caries
2 = Filled w/caries 3 = Filled, no caries 4 = Missing due to caries
5 = Missing for any another reason
6 = Fissure sealant
7 = Fixed dental prosthesis/ crown abutment, veneer, implant
8 = Unerupted
9 = Not recorded

Bleeding 109 (124)

| 18 | 17 | 16 | 15 | 14 | 13 | 12 | 11 | 21 | 22 | 23 | 24 | 25 | 26 | 27 | 28 |
|---|---|---|---|---|---|---|---|---|---|---|---|---|---|---|---|
| | | | | | | | | | | | | | | | |
| | | | | | | | | | | | | | | | |

Bleeding 141 (156)

| | | | | | | | | | | | | | | | |
|---|---|---|---|---|---|---|---|---|---|---|---|---|---|---|---|
| | | | | | | | | | | | | | | | |
| | | | | | | | | | | | | | | | |

**Gingival bleeding**

**Score**
0 = Absence of condition
1 = Presence of condition
9 = Tooth excluded
X = Tooth not present

**Pocket**

**Score**
0 = Absence of condition
1 = Pocket 4–5 mm
2 = Pocket 6 mm or more
9 = Tooth excluded
X = Tooth not present

**Oral mucosal lesions**

☐ (186) ☐ (187) ☐ (188)

(189) ☐ (190) ☐ (191) ☐

**Condition**

0 = No abnormal condition
1 = Malignant tumour (oral cancer)
2 = Leukoplakia
3 = Lichen planus
4 = Ulceration (aphthous, herpetic, traumatic)
5 = Acute necrotizing ulcerative gingivitis (ANUG)
6 = Candidiasis
7 = Abscess
8 = Other condition (specify if possible)
9 = Not recorded

**Location**

0 = Vermillion border
1 = Commissures
2 = Lips
3 = Sulci
4 = Buccal mucosa
5 = Floor of the mouth
6 = Tongue
7 = Hard and/or soft palate
8 = Alveolar ridges/gingiva
9 = Not recorded

**Denture(s)**

| **Upper** | **Lower** |
|---|---|
| ☐ (192) | ☐ (193) |

**Status**

0 = No denture
1 = Partial denture
2 = Complete denture
9 = Not recorded

**Intervention urgency** ☐ (194)

0 = No treatment needed
1 = Preventive or routine treatment needed
2 = Prompt treatment (including scaling) needed
3 = Immediate (urgent) treatment needed due to pain or infection of dental and/or oral origin 4 = Referred for comprehensive evaluation or medical/dental treatment (systemic condition)

Oral Hygiene Index Status

DI-S = _________

| 16B | 11La | 26B |
|---|---|---|
| | | |
| | | |
| 46Li | 31La | 36Li |

CI-S = _________

| 16B | 11La | 26B |
|---|---|---|
| | | |
| | | |
| 46Li | 31La | 36Li |

OHI-S = _________________
